# Mutation spectrum of *CYP1B1* and *MYOC* genes in Korean patients with primary congenital glaucoma

**Published:** 2011-08-09

**Authors:** Hee-Jung Kim, Wool Suh, Sung Chul Park, Chan Yun Kim, Ki Ho Park, Michael S. Kook, Yong Yeon Kim, Chang-Sik Kim, Chan Kee Park, Chang-Seok Ki, Changwon Kee

**Affiliations:** 1 Department of Laboratory Medicine & Genetics, Samsung Medical Center, Sungkyunkwan University School of Medicine, Seoul, Korea; 2 Department of Ophthalmology, Samsung Medical Center, Sungkyunkwan University School of Medicine, Seoul, Korea; 3 Department of Ophthalmology, Yonsei University College of Medicine, Seoul, Korea; 4 Department of Ophthalmology, Seoul National University College of Medicine, Seoul, Korea; 5 Department of Ophthalmology, College of Medicine, University of Ulsan, Asan Medical Center, Seoul, Korea; 6 Department of Ophthalmology, Korea University College of Medicine, Seoul, Korea; 7 Department of Ophthalmology, College of Medicine, Chungnam National University, Daejon, Korea; 8 Department of Ophthalmology and Visual Science, Seoul St. Mary’s Hospital, The Catholic University of Korea School of Medicine, Seoul, Korea

## Abstract

**Purpose:**

To elucidate the incidence of cytochrome P450 1B1 (*CYP1B1*) and myocillin (*MYOC*) mutations in Korean patients with primary congenital glaucoma (PCG).

**Methods:**

Genomic DNA was collected from peripheral blood of 85 unrelated Korean patients who were diagnosed as having PCG by standard ophthalmological examinations and screened for mutations in the *CYP1B1* and *MYOC* genes by using bi-directional sequencing.

**Results:**

Among 85 patients with PCG, 22 patients (22/85; 25.9%) had either one (n=11) or two (n=11) mutant alleles of the *CYP1B1* gene. Among 11 different* CYP1B1* mutations identified, a frameshift mutation (c.970_971dupAT; p.T325SfsX104) was the most frequent mutant allele (6/33; 18.2%) while p.G329S and p.V419Gfs11X were novel. In the *MYOC* gene, two variants of unknown significance (p.L228S and p.E240G) were identified in two PCG patients (2/85; 2.4%), respectively. No patient had mutations in both genes.

**Conclusions:**

Although *CYP1B1* mutations are major causes of PCG in Korea, ~70% of PCG patients have neither *CYP1B1* nor *MYOC* mutations suggesting a high degree of genetic heterogeneity. Furthermore, the fact that 11 out of 22 patients had only one mutant allele in the *CYP1B1* gene necessitates further investigation for other genetic backgrounds underlying PCG.

## Introduction

Primary congenital glaucoma (PCG; OMIM 231300) is a rare but severe form of glaucoma, which usually manifests within the first year of life [1]. It is characterized by high ocular pressure (IOP) resulting from an obstruction of aqueous outflow from the anterior segment of the eye, and is thought to be the result of an anatomic defect in the trabecular meshwork and anterior chamber [2]. Increased IOP causes irreversible damage to the optic nerve and can lead to blindness if untreated. Affected children typically present with photophobia, epiphora, corneal clouding, and enlargement of globe or cornea. PCG occurs in both familial and sporadic patterns [3]. Inheritance in familial cases is usually autosomal recessive. Incidence of PCG is geographically and ethnically variable, with the lowest incidence (1:10,000) in the Western population and higher incidence in inbred populations, such as the Gypsy subpopulation of Slovakia (1:1,250) [4]. Three loci have been mapped for PCG (gene symbol GLC3), GLC3A (2p21; OMIM 231300), GLC3B (1p36.2; OMIM 600975), and GLC3C (14q24.3). The gene associated with GLC3A, cytochrome P450, family 1, subfamily B, polypeptide 1 (*CYP1B1*; OMIM 601771), has been implicated in the pathogenesis of PCG. Physiologic studies have confirmed that mutations in *CYP1B1* can cause disease; however, the pathway by which *CYP1B1* affects development of the anterior chamber of the eye is unknown. The proportion of PCG patients whose disease is due to *CYP1B1* mutations is generally high, but varies among populations, ranging from 100% in Slovakian Roma to ~10% in Mexico [4,5]. No responsible gene has yet been identified at the GLC3B and GLC3C loci [6].

Of particular interest, the myocilin gene (*MYOC*; OMIM 601652), the first open angle glaucoma gene, was initially reported to interact with *CYP1B1* through a digenic mechanism, leading to juvenile open angle glaucoma [7]. However, *MYOC* has recently been implicated in the pathogenesis of some cases of PCG, either independently or in association with *CYP1B1* [8,9].

Herein, we screened both the *CYP1B1* and *MYOC* genes in 1 familial and 84 sporadic cases of PCG to identify the underlying genetic mutations in a Korean population.

## Methods

### Subjects

The study protocol adhered to the tenets of the Declaration of Helsinki and informed consent was obtained from patients or their responsible guardians. We conducted a prospective multi-institutional collaborative study from September, 2008 to February, 2010. A total of 85 unrelated PCG patients were recruited from seven hospitals in South Korea. Of the 85 cases, only one case was familial and the rest were sporadic. Criteria for PCG diagnosis included IOP ≥21 mmHg in at least one eye; megalocornea; corneal edema/clouding/ opacity; and glaucomatous optic nerve head damage when examination was possible. Corroborating features included symptoms of epiphora and photophobia. Patients with other ocular or systemic anomalies were excluded.

To determine whether sequence variants identified were polymorphic in Korean population, we used a panel of DNA from 105 to 200 unrelated Korean individuals who attended the clinic for conditions other than glaucoma.

### Genetic analyses of *CYP1B1* and *MYOC* genes

Blood samples were taken from affected subjects and their parents or relatives when possible. According to the manufacturer’s instructions, genomic DNA was isolated from peripheral blood leukocytes using the Wizard genomic DNA purification kit (Promega, Madison, WI). Using the primers designed by the authors (Table 1 and Table 2), entire coding exons and flanking intronic sequences of *CYP1B1* and *MYOC* were amplified by polymerase chain reaction (PCR). Using the BigDye Terminator Cycle Sequencing Ready Reaction kit (Applied Biosystems, Foster City, CA), cycle sequencing of *CYP1B1* and *MYOC* was performed on the ABI 3100 Genetic Analyzer (Applied Biosystems).

**Table 1:** PCR and sequencing primers for *CYP1B1* gene analysis.

**Primer name**	**Primer sequence (5'→3')**	**Size (bp)**
CYP1B1 1F	TCTCCAGAGAGTCAGCTCCG	786
CYP1B1 1R	GGGTCGTCGTGGCTGTAG	
CYP1B1 2F	ATGGCTTTCGGCCACTACT	787
CYP1B1 2R	GATCTTGGTTTTGAGGGGTG	
CYP1B1 3F	AGTGAGAAATTAGGAAGCTGTTTTAGA	594
CYP1B1 3R	GCCAGGATGGAGATGAAGAG	
CYP1B1 4F	CCCAAGGACACTGTGGTTTT	498
CYP1B1 4R	AACGCTAATTGAGAAGCAGCA	

Annealing temperature, 60 °C for all primer pairs.

**Table 2:** PCR and sequencing primers for *MYOC* gene analysis.

**Primer name**	**Primer sequence (5'→3')**	**Size (bp)**
MYOC 1F	CTCTGTCTTCCCCCATGAAG	462
MYOC 1R	AGCCTGGTCCAAGGTCAAT	
MYOC 2F	AGGCCATGTCAGTCATCCAT	478
MYOC 2R	GCGCCTGTAGCAGGTCACTA	
MYOC 3F	GCAGCCTATTTAAATGTCATCCT	310
MYOC 3R	TGGGTGGGCATTTACCCTAT	
MYOC 4F	TCCGCATGATCATTGTCTGT	467
MYOC 4R	ACCCCAAGAATACGGGAACT	
MYOC 5F	ACTCGGGGAGCCTCTATTTC	461
MYOC 5R	CTCCAGGGGGTTGTAGTCAA	
MYOC 6F	CCCAGAGAATCTGGAACTCG	478
MYOC 6R	CGCCCTCAGACTACAATTCC	

Annealing temperature, 60 °C for all primer pairs.

The Sequencher program (Gene Codes Corp., Ann Arbor, MI) was used for analysis of sequence variations with reference to the wild type sequence. Variations were described according to guidelines established by the Human Genome Variation Society (HGVS); the ‘A’ of the ATG codon for translation initiation was numbered +1 and the 1st methionine was numbered +1 (CYP1B1; NP_000095.2, MYOC; NP_000252.1). We referred to the *CYP1B1* and *MYOC* mutation database, as well as the literature, to determine whether a detected variation was novel or known [10]. When a novel sequence variant was identified, 400 ethnically matched normal control chromosomes were tested for the presence of the variant.

To assess the extent of conservation of a novel variation in *CYP1B1* thought to be associated with disease, the deduced amino acid sequence was assessed by aligning the protein sequences of different mammalian species and of related *CYP1B1* family members using ClustalW2 software (European Bioinformatics Institute, Hinxton, UK).

## Results

### Mutational analysis of *CYP1B1*

**Figure 1:**
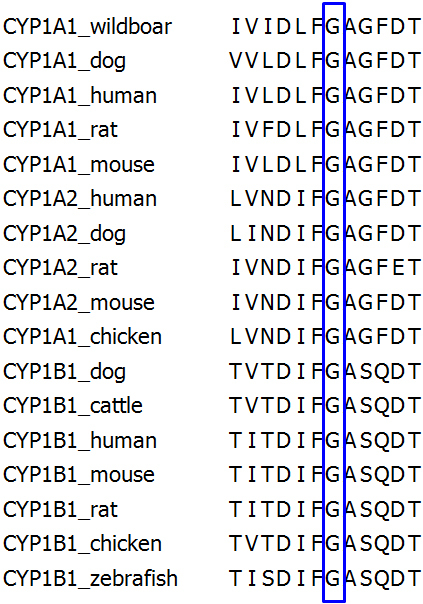
Conservation of p.Gly329 residues (numeration according to human *CYP1B1*, as shown by protein alignment of several *CYP1B1* orthologs and other CYP family members, using ClustalW2.

**Figure 2:**
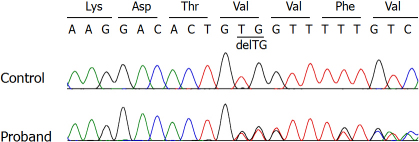
Direct sequencing of the *CYP1B1* gene. p.V419GfsX11 is a novel deletion mutation detected in a patient in the compound heterozygous state.

Eleven different mutations in 22 sporadic cases were identified by direct sequencing of the *CYP1B1* gene in 85 PCG probands (Table 3). In total, three subjects were homozygous for a *CYP1B1* mutation and 8 patients were compound heterozygous. Another 11 patients were heterozygous for a *CYP1B1* mutation. Among 11 different mutations, two novel variations, including a missense variation and a 2-bp deletion (p.G329S and p.V419Gfs11X), were identified. The p.G329S variation occurs in a highly conserved residue (Figure 1) and p.V419Gfs11X causes a frameshift, resulting in a premature termination codon at residue 429 (Figure 2). These two variations were not found in 400 normal chromosomes. The remaining 9 mutations identified in our cohort have been reported previously. They included the following missense changes: c.985G>T (p.V320L), c.988_989delinsTT (p.A330F), c.1090G>A (p.V364M), c.1103G>A (p.R368H), c.1169G>A (p.R390H), and c.1331G>A (p.R444Q), along with three deleterious mutations, including c. 55C>T (p.Q19X), c.243C>G (p.T81X), and a two base pair duplication in exon 2 (c.970_971dupAT; p.T325SfsX104). p.T325SfsX104 was the most frequent allele (18.2%, 6/33) and exhibited compound heterozygosity with p.T81X, p.G329S, p.R390H, and p.R444Q, along with one case of homozygosity (Table 3).

**Table 3:** *CYP1B1* mutations identidied in Korean probands with PCG.

**Patients**	**Nucleotide change**	**Amino acid change**	**Hetero-/homozygous**
PCG 11	c.[243C>G]+[1090G>A]	p.[Y81X]+[V364M]	Compound heterozygous
PCG 17	c.[1090G>A]+[1090G>A]	p.[V364M]+[V364M]	Homozygous
PCG 18	c.[958G>T]+?	p.[V320L]+[?]	Heterozygous
PCG 20	c.[1090G>A]+?	p.[V364M]+[?]	Heterozygous
PCG 37	c.[958G>T]+?	p.[V320L]+[?]	Heterozygous
PCG 40	c.[970_971dupAT]+**[985G>A]**	p.[T325SfsX104]+**[G329S]**	Compound heterozygous
PCG 45	c.[55C>T]+[1103G>A]	p.[Q19X]+[R368H]	Compound heterozygous
PCG 46	c.1090G>A+?	p.[V364M]+[?]	Heterozygous
PCG 49	c.[243C>G]+[970_971dupAT]	p.[Y81X]+[T325Sfs104X]	Compound heterozygous
PCG 53	c.[958G>T]+?	p.[V320L]+[?]	Heterozygous
PCG 54	c.[988_989delGCinsTT]+[1256_1257delTG]	p.[A330F]+**[V419GfsX11]**	Compound heterozygous
PCG 55	c.[958G>T]+?	p.[V320L]+[?]	Heterozygous
PCG 6	c.[970_971dupAT]+[970_971dupAT]	p.[T325SfsX104]+[T325SfsX104]	Homozygous
PCG 69	c.[988_989delGCinsTT]+[1331G>A]	p.[A330F]+[R444Q]	Compound heterozygous
PCG 72	c.[988_989delGCinsTT]+?	p.[A330F]+[?]	Heterozygous
PCG 73	c.[958G>T]+?	p.[V320L]+[?]	Heterozygous
PCG 74	c.[988_989delGCinsTT]+?	p.[A330F]+[?]	Heterozygous
PCG 75	c.[970_971dupAT]+[1331G>A]	p.[T325SfsX104]+[R444Q]	Compound heterozygous
PCG 78	c.[970_971dupAT]+[1169G>A]	p.[T325SfsX104]+[R390H]	Compound heterozygous
PCG 84	c.[988_989delGCinsTT]+?	p.[A330F]+[?]	Heterozygous
PCG 86	**c.[985G>A]**+**[985G>A]**	**p.[G329S]**+**[G329S]**	Homozygous
PCG 100	**c.[985G>A]**+[?]	**p.[G329S]**+[?]	Heterozygous

*Reported as a causative mutation for hepatocellular adenoma. **Bold** lettering is used to represent novel mutations identified in this study.

Eleven probands had only one identifiable mutant allele. The mutations found in the heterozygous state were p.V320L, p. A330F, p.V364M, and p.G329S.

In addition to 11 different mutations, 7 previously reported polymorphisms, c.-13C>T, c.142C>G (p.R48G), c.319C>G (p.L107V), c.355G>T (p.A119S), c.729G>C (p.V243V), c.1294G>C (p.V432L), and c.1347T>C (p. D449D) were detected (Table 4).

**Table 4:** Single nucleotide polymorphisms of *CYP1B1* identified in Korean probands with PCG.

**Location**	**Nucleotide change**	**Amino acid change**	**Allele frequency (%)**		**Reference SNP number**
Intron 1	5′UTR-13C>T	NA	C 130/170 (76.5)	T 40/170 (23.5)	rs2617266
Exon 2	c.142C>G	p.R48G	C 130/170 (76.5)	G 40/170 (23.5)	rs10012
Exon 2	c.319C>G	p.L107V	C 166/170 (97.6)	G 4/170 (2.4)	rs56339482
Exon 2	c.355G>T	p.A119S	G 129/170 (75.9)	T 41/170 (24.1)	rs1056827
Exon 2	c.729G>C	p.V243V	G 165/170 (97.1)	C 5/170 (2.9)	rs9341249
Exon 3	c.1294G>C	p.V432L	G 23/170 (13.5)	C 147/170 (86.5)	rs1056836
Exon 3	c.1347T>C	p.D449D	T 27/170 (15.9)	C 143/170 (84.1)	rs1056537

Abbreviations: SNP, single nucleotide polymorphism; UTR, untranslated region; NA, not applicable.

### Mutational analysis of *MYOC*

Due to DNA availability, direct sequencing of *MYOC* was performed for 79 out of 85 patients. As a result, 2 patients were shown to harbor possible novel mutations in *MYOC*: c.683T>C (p.L228S) and c.719A>G (p.E240G), respectively. Neither of the two variations was found in 400 normal chromosomes. Five previously described polymorphisms, including c.34G>C (p.G12R), c.227G>A (p.R76K), c.624C>G (p.D208E), c.730+35G>A, and c.1058C>T (p.T353I) were identified (Table 5). In addition, two novel synonymous variations, c.864C>T (p.I288I) and c.1110G>A (p.P370P), were each found once in heterozigosity (0.6%, 1/158). These two variations were not found in 400 normal chromosomes.

**Table 5:** Single nucleotide polymorphisms of *MYOC* in korean patients with PCG.

**Location**	**Nucleotide change**	**Amino acid change**	**Allele frequency (%)**				**Reference SNP number**
			**PCG (n=79)**		**Control (n=200)**		
Exon1	c.34G>C	p.G12R	G 2/158 (1.3)	C 156/158 (98.7)	G 395/400 (98.8)	C 5/400 (1.3)	Rare polymorphism [32,33],
Exon1	c.227G>A	p.R76K	G 8/158 (5.1)	A 150/158 (94.9)	G 383/400 (95.8)	A 17/400 (4.3)	rs2234926
Exon2	c.624C>G	p.D208E	C 2/158 (1.3)	G 156/158 (98.7)	C 391/400 (97.8)	G 9/400 (2.3)	rs2234927
Intron2	IVS2+35G>A	NA	G 10/158 (6.3)	A 148/158 (93.7)	G 71/400 (17.8)	A 329/400 (82.3)	rs2032555
Exon3	**c.864C>T**	**p.I288I**	C 1/158 (0.6)	T 157/158 (99.4)	C 400/400 (100.0)	T 0/400 (0.0)	This study
Exon3	c.1058C>T	p.T353I	C 1/158 (0.6)	T 157/158 (99.4)	C 399/400 (99.8)	T 1/400 (0.3)	Rare polymorphism [33,36,38],
Exon3	**c.1110G>A**	**p.P370P**	G 1/158 (0.6)	A 157/158 (99.4)	G 400/400(100.0)	A 0/400 (0.0)	This study

Abbreviation: SNP, single nucleotide polymorphism; NA, not applicable. **Bold** lettering is used to represent novel variations in this study.

## Discussion

Herein, we report on mutation screening of the *CYP1B* and *MYOC* genes in 85 and 79 PCG cases, respectively. *CYP1B1* screening revealed that about 26% of 85 patients had at least one mutation, although half of them carried only one mutant allele with the other mutation unidentified. Homozygisity of the mutant allele was seen in only three cases and compound heterozygosity in eight cases. Consistent with other *CYP1B1* mutation spectrum studies from populations where consanguinity is uncommon, we observed a high degree of allelic heterogeneity and compound heterozygosity. To the best of our knowledge, of 11 different *CYP1B1* mutations identified in the 85 probands, two are novel.

p.G329S has been previously reported as a pathologic mutation for hepatocellular carcinoma [11]; however, in this study, it was reported as a new causative mutation for PCG in a case of compound heterozygosity with p.T325SfsX104 along with one each case of homozygosity and heterozygosity. p.G329S occurs at an amino acid position that is highly conserved among other species and *CYP1B1* family members (Figure 1).

Among all of the mutations, p.T325SfsX104 was most frequently found (6/33 mutant alleles). It is interesting to note that this disease-causing mutation has only been described in a single Japanese patient with PCG [12]. We speculated that p.T325SfsX104 might be recurrent among Korean individuals with PCG, possibly with a founder effect.

Although the presence of heterozygous *CYP1B1* mutations in 11 PCG patients does not match a typical recessive pattern of inheritance in PCG, heterozygous *CYP1B1* mutations have been documented [13-17]. Mutations such as p.T81N, p.Q229K, p.R368H, and p.R469W have been described in PCG patients in the heterozygous state [13,15-18]. The mutations found in the heterozygous state in this study were p.V320L, p.A330F, p.V364M, and p.G329S. As described above, p.G329S is a novel mutation. Since p.V320L, p.A330F, and V364M, have not been reported in heterozygous state [12,19-22], additionally, we performed population screening involving 210 control chromosomes. As a result, p.A330F and p.V364M were not found in any normal chromosome and p.V320L was occurred in less than 1% (2/210). As only the coding region of *CYP1B1* was sequenced, we thought that it might be due to mutations in (1) the *CYP1B1* promoter or other non-coding regions; (2) genes linked to other PCG loci, such as GLC3B and GLC3C; (3) other glaucoma genes such as *MYOC*, resulting in digenic inheritance; or (4) some other unknown genes causing glaucoma. The presence of double heterozygous variants, *CYP1B1* and *MYOC*, has recently been described in one PCG case; however, the role of possible digenism in disease causation is yet to be established [8,23]. PCG-causing mutations in latent transforming growth factor beta binding protein 2 (*LTBP2*; OMIM 602091) have recently been identified in Pakistani, European Gypsy, and Iranian patients [24,25]. *LTBP2* lies very close to GLC3C on chromosome 14, but is not strictly within the locus, as originally defined [26]. As such, it is not clear whether *LTBP2* is the PCG-associated gene within GLC3C, or whether the gene within this locus remains unknown and *LTBP2* defines a fourth locus for PCG. Observation of unrelated PCG cases with a heterozygous mutation also raises the possibility that the mutation might be a dominant cause of PCG [16].

Due to ethnic differences and geographical variations, the prevalence of *CYP1B1* mutations varies in different patient populations, from ~10% in Mexico [5], to 20% in Indonesia [27], Australia [28], China [29], and Japan [19]; around 40% in Turkish patients [13]; approximately 50% in Brazil [20] and France [16]; and about 100% in consanguineous Saudi Arabian [30] and Slovakian Gypsy [4] patients. Although direct comparison between these studies is difficult, it should be noted that the proportion of *CYP1B1* mutations accounting for PCG in our population is similar to those in Japan and China. These data also illustrate that the contribution of defects in this gene varies significantly among human populations, which highlights the need for analysis of large groups of PCG from different ethnic backgrounds to ascertain the role of this gene in a specific population.

**Figure 3:**
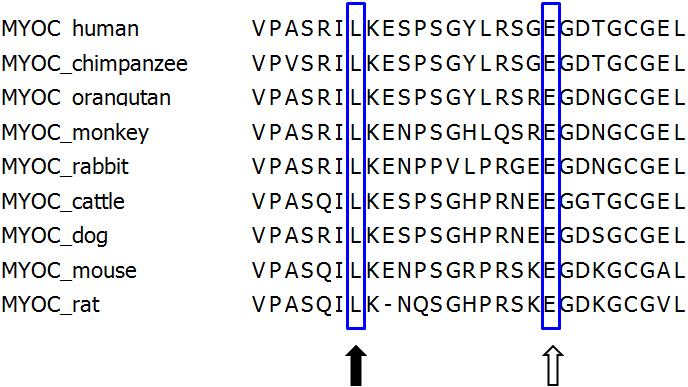
Alignment of *MYOC* peptide sequences in human and other species. The CLUSTAL W (2.0) computer program was used for multiple alignment of amino acid sequences. The position of mutated amino acids in the PCG patient reported in this study is boxed. Note that the p.L228S and p.E240G mutations occurred at highly conserved positions of the amino acids (filled arrow; p.Leu228, open arrow; p.Glu240).

Direct sequencing of the coding region of *MYOC* was performed in 79 of all PCG probands. As a result, two variants of unknown significance (p.L228S and p.E240G) were identified in two PCG patients (2/79; 2.5%). These variations lead to replacement of leucine by serine at codon 228 (p.L228S) and glutamic acid by glycine at codon 240 (p.E240G), respectively, which involve a highly conserved region among other species (Figure 3). Neither p.L228S nor p.E240G was found in 400 control chromosomes, suggesting that these two variations are possible mutations. In one previous study, *MYOC* mutations accounted for 2.6% (3/116) of Chinese patients with PCG [29].

Of the polymorphisms identified, p.G12R and p.T353I have been previously reported as possible POAG causing mutations; however, they were found in control subjects in this study (Table 5). p.G12R, which has been reported as a possible disease-causing mutation in a sporadic northern Chinese case of POAG [31], was found in control subjects in a few studies [32,33]. p.T353I was found in a Korean family with POAG [34], a Japanese patient with POAG [35], seventeen Chinese individuals with POAG [31-33,36], and one Indian patient with juvenile-onset POAG [37]. However, this change has been also detected in normal individuals [33,36,38]. Therefore, p.G12R and p.T353I are rare polymorphisms rather than disease-causing mutations; however, it remains possible that they affect the risk of POAG.

To the best of our knowledge, this is the first report on molecular genetic analysis of PCG in the Korean population. One fourth of Korean PCG probands were found to have at least one *CYP1B1* mutation and half of the patients with *CYP1B1* mutations had only one mutant allele. We observed a high degree of allelic heterogeneity in our cohort with *CYP1B1* mutations. Only two patients carried possible *MYOC* mutations. These results suggest that *CYP1B1* should be regarded as a potential primary cause of PCG in Korea. However, due to the relatively low contribution of CYP1B1 to Korean PCG, it is suggested that other genetic factors remain to be identified and that further work is needed to identify the causative genes.
